# Supplementary material for: Healthcare considerations for aging people in prisons
Source: PLoS One. 2026 Apr 30;21(4):e0347851. doi: 10.1371/journal.pone.0347851 (PMC13132422; doi:10.1371/journal.pone.0347851)
Supplement: S1 Text — (PDF) [file pone.0347851.s001.pdf]

IJPH | SSPH+ SWISS SCHOOL OF PUBLIC HEALTH

International Journal of Public Health
THEORY & CONCEPT
published: 01 August 2024
doi: 10.3389/ijph.2024.1607253

A Process Model of Formative Work to Strengthen a Prison Health Surveillance System

Jessica Gaber, Njideka Sanya, Jennifer Lawson, Iridian M. Grenada and Fiona G. Kouyoumdjian*

Department of Family Medicine, McMaster University, Hamilton, Canada

Worldwide, there is a lack of systematically collected health data on people who are incarcerated. Our objective in this paper was to describe a process model of formative work for a project to strengthen health surveillance for people incarcerated under a Canadian prison authority. We have developed project structures and processes, and we are evaluating project partnerships. To inform prison health surveillance foci, we are conducting a review of literature on best practices, a qualitative study to understand stakeholders' needs and priorities, and mapping work to understand available prison health-related data. Developing and implementing prison health surveillance is gradual and developmental, necessitating time to build relationships and obtain approvals. The needs and interests of knowledge users should be prioritized, but there may be challenges to achieving a coherent vision due to feasibility and differing needs and objectives of various stakeholders. Developing collaborative relationships could help bridge this gap.

Keywords: prison health, process model, health surveillance, prisons, literature review

OPEN ACCESS

Edited by:
Nino Kuenzli,
Swiss Tropical and Public Health Institute (Swiss TPH), Switzerland

Reviewed by:
Marlene Joannie Bewa,
University of South Florida, United States
One reviewer who chose to remain anonymous

****Correspondence***
Jessica Gaber,
jgaber@mcmaster.ca

Received: *07 March 2024*
Accepted: *19 July 2024*
Published: *01 August 2024*

Citation:
Gaber J, Sanya N, Lawson J, Grenada IM and Kouyoumdjian FG (2024) A Process Model of Formative Work to Strengthen a Prison Health Surveillance System. Int J Public Health 69:1607253. doi: 10.3389/ijph.2024.1607253

INTRODUCTION

Despite the large number of people who experience incarceration worldwide and substantial evidence regarding the poor health status of this population, there is insufficient high quality data on the health of incarcerated people globally [1]. This may be due in part to the complex ethical, logistical, and legal challenges associated with data collection, use, and reporting [2, 3]. In particular, there has been a lack of attention to the development of health surveillance, "the ongoing systematic collection, analysis, interpretation, and dissemination of health data for the planning, implementation, and evaluation of public health action" [4] in correctional settings [5–7]. A review of prison health monitoring systems in the early 2010s found that of 15 high-income countries studied, only four had developed a long-term general monitoring system: two (including Canada) collected prisoners' health data in a computerized system and two conducted regular nationwide surveys, and only data on mortality were routinely collected across all these jurisdictions [8] notably, such data collection represents only the first step of health surveillance, and additional work of data analysis, interpretation, and reporting would be required to constitute effective health surveillance.

Further, gaps in health data for people who are incarcerated result from often being excluded from national surveillance systems and health administrative data, contributing to a lack of knowledge, and specifically precluding comparisons with data for others in the population. Most national public health surveillance systems do not include data for people who are incarcerated [8, 9], for example, national surveys in Canada typically explicitly exclude institutionalized populations [10], which

includes people in prison. The administrative structure of healthcare and correctional health data may also impact the ability to collect and compare data. For example, in Canada, data from healthcare encounters in federal prisons (which are administered by the federal government) as well as some healthcare data from provincial and territorial prisons are not included in many provincial and territorial health administrative datasets, which are often used to assess population health and healthcare system performance. These exclusions of people and data prevent the use of data for important surveillance purposes.

As in other healthcare and community settings, prison health surveillance data could be used to tailor programs and policies, for example to improve health promotion, health protection, disease and injury prevention, and healthcare. In prisons, health surveillance should also be used for information specific to this setting, for example to understand and communicate about the prison context, the experience of incarceration, and specific aspects of the population health status of people who are incarcerated, including health equity, human rights, and legal considerations [3, 11, 12].

With these important needs and purposes in mind, we undertook a project to strengthen health surveillance in a Canadian prison authority. The Canadian Correctional Health Information for ActioN Group Endeavour, or C-CHANGE (pronounced "sea change") represents a new approach to working together to conduct health surveillance. The project represents a partnership with collaboration and engagement of an academic partner, a prison authority, and community partners. C-CHANGE is supported by funding from the Canadian federal government obtained through a competitive grant focused on health surveillance. As articulated in the funded proposal, the primary project focus was initially on facilitating participation of the prison authority in a national health surveillance system that leverages electronic health record (EHR) data from routine healthcare encounters, and the project has developed to include other activities to enhance surveillance based on partners' interests and identified opportunities. In this paper, our objective is to describe a process model of formative work for this project on prison health surveillance.

METHODS

Setting and Context

This project is focused on health surveillance in a Canadian prison authority. This prison authority administers various types of correctional facilities (e.g., prisons, correctional centres, healing lodges) with varying security levels (e.g., maximum, medium, minimum), which are located across the country. Available data indicate an average cross-sectional population in custody of 12,394 in 2021/2022, down from over 14,000 people in the three pre-pandemic years [13], and a much larger population would experience incarceration over a period of years as new people are incarcerated while others are released over time; for example, there are approximately seven thousand people released each year from federal custody, about one-third of whom are Indigenous [14]. The prison authority has responsibility for and oversight of health surveillance for people who are incarcerated in the correctional system, and has been developing its health surveillance over many years.

The Process Model

The process model of this formative work includes inputs of both people and activities, project structures that manage implementation of the prison health surveillance system, and considerations that span project-wide. **Figure 1** shows a high-level conceptual model for project design including structures, processes, and key activities. These described in further detail below.

Stakeholder Engagement

As the academic partner, we lead project planning, coordination, and implementation. The prison authority provides input into project activities and is a key knowledge user for all project outputs, as the authority will decide how to integrate outputs and information from all project activities into their ongoing surveillance. The prison authority also leads components of work related to participation in the national surveillance system using EHR data, including technical work, assessment of privacy and data security, and obtaining approvals.

The Project Implementation Team involves the academic partner, the prison authority, and the national surveillance system. This team leads work to support participation in the national surveillance system using EHR data. The Information Technology (IT)/Data Management Team focuses on technical work including work to use evidence-based algorithms to identify people with specific health conditions and to enable participation in the national surveillance system, and provides input to the Project Implementation Team about that work. The Project Advisory Committee includes people with lived experience of incarceration and representatives of community organizations, and provides advice regarding the project workplan as well as specific practical and ethical issues to support project implementation, for example, strategies for research recruitment and considerations regarding risks involved in data collection for health surveillance. The Scientific/Academic Team includes people with academic expertise in prison health and health surveillance, among other areas, who contribute to the scholarly components of work, including research activities. Through these structures and established processes, the academic partner, the prison authority, the Project Advisory Committee, and the Scientific/Academic team all influence and contribute to ongoing project activities.

Project Evaluation and Ethical Considerations

Across all project activities, we have attended to evaluation of the partnership and project, privacy and confidentiality, and legal and ethical issues. As required by the project funder (as a standard component for this grant opportunity), we are conducting ongoing evaluation of the project and project partnerships with an independent third-party evaluator, which supports project implementation and the development of ongoing partnership and collaboration. We engage in discussions

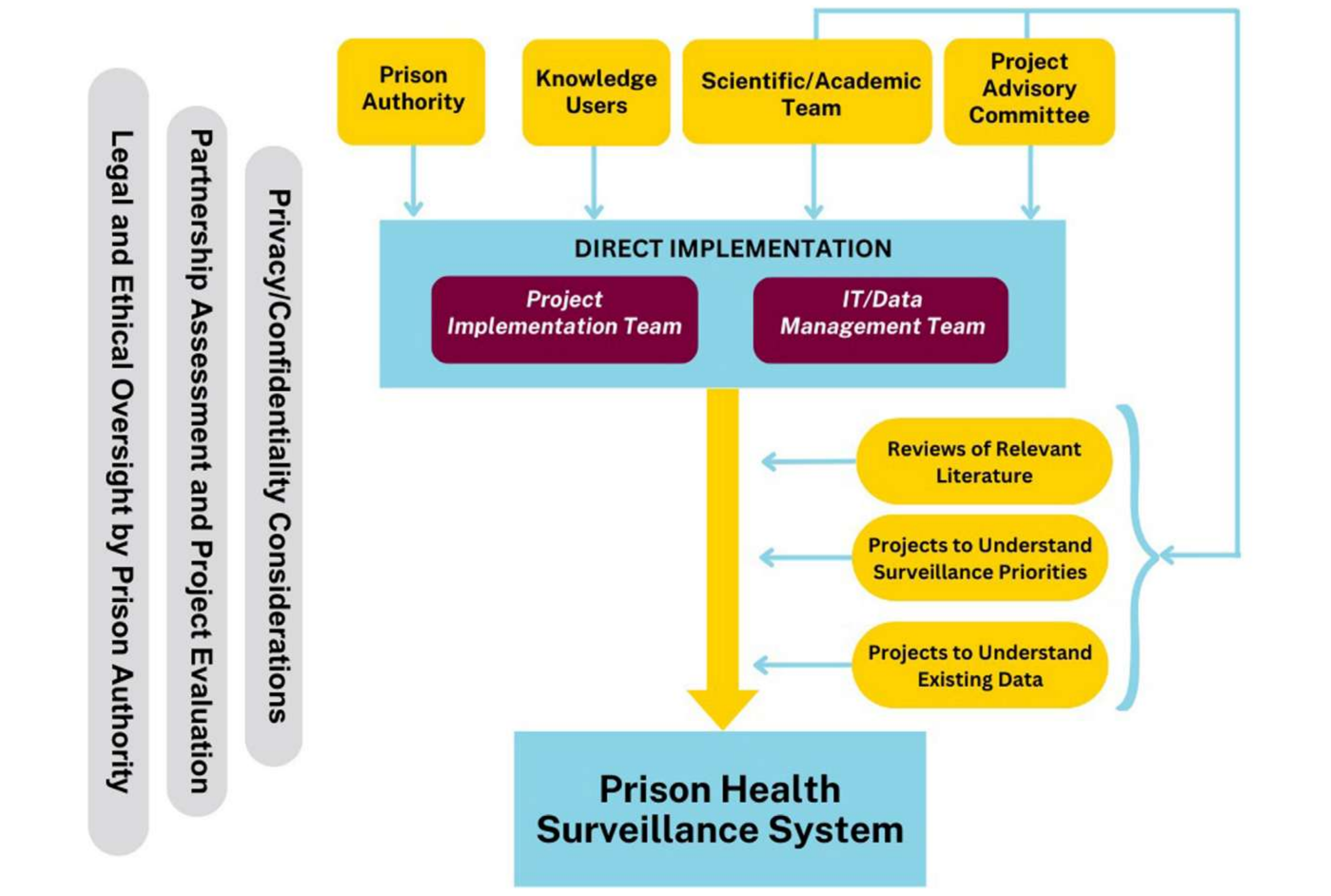

FIGURE 1 | Conceptual Model of the Canadian Correctional Health information for Action Group Endeavour (C-CHANGE) Process, Canada, 2021–2024.

regarding privacy and confidentiality with respect to data collection in prisons with the Project Advisory Committee, and with respect to data sharing and data access with the Project Implementation Team. The prison authority has legal and ethical oversight of health surveillance, but in all project structures we engage in discussions regarding ethical issues associated with health surveillance.

C-CHANGE represents an implementation science project rather than research project, so the overall project did not require ethics review. One project component was a research project to understand stakeholders' prison health surveillance needs and priorities (described below), and we obtained institutional ethics approval for this research study (Hamilton Integrated Research Ethics Board, project #14099).

Formative Research

We are developing other inputs to inform an emerging vision of health surveillance for the prison authority. Three key initiatives are a review of academic literature on best practices for prison health surveillance, the aforementioned research with key stakeholders to define prison health surveillance needs and priorities, and mapping work to understand available health-related data in the prison authority.

Review of Academic Literature on Best Practices for Prison Health Surveillance

We reviewed the scientific literature on prison health surveillance to inform our conceptualization of best practices and roles for surveillance. Two recent articles provide useful frameworks for strengthening prison health surveillance.

First, Binswanger et al. described the importance of collecting national health data for people who are incarcerated to meet the needs of healthcare providers, policymakers, and the public [3]. They identified public health surveillance as one of six potential purposes of collecting health data on people who are incarcerated, alongside health promotion and disease prevention, healthcare performance and patient value, policy relevance, health equity, and human rights and legal considerations. They also described five fundamental principles of prison health data collection: "1) ensure reliable and valid data collection and measurement, 2) use measures of evidence-based health practices, 3) align measures and approaches with general population data collection and across regions and settings, 4) promote transparency while maintaining trust and ensuring individual privacy, and 5) encourage greater patient-centeredness" (p. 40S).

Second, Perrett et al. proposed the Five Nations model as a useful model for prison health surveillance system design and

evaluation [12]. The model builds on six indicators of public health surveillance system usefulness from the U.S. Centers for Disease Control and Prevention [11], which are to estimate the magnitude of morbidity and mortality; identify trends for diseases, injury, exposure, or outbreaks; support timely and accurate detection of diseases, injuries, or outbreaks; assess prevention and control programs; lead to improved clinical, behavioural, social, policy, or environmental practices; and stimulate research related to prevention or control. The Five Nations model adds four features that reflect the needs of the prison setting: data collection on population size and demographics, the communities from which the prison population comes and to which they return, the impact of the physical environment (e.g., availability and uptake of exercise, education, purposeful activity, social visits, time out of cell, and closed or open prison conditions), and the interdependency of health and justice services on wellbeing (e.g., levels of under/overcrowding, staffing levels, security of estate, availability of contraband, availability of services addressing offending behavior, and pastoral support).

In addition, we reviewed United Nations documents, i.e., the *United Nations Standard Minimum Rules for the Treatment of Prisoners* (The Nelson Mandela Rules) [15]and the *United Nations Rules for the Treatment of Women Prisoners and Non-Custodial Measures for Women Offenders* (The Bangkok Rules) [16] and we used the content of these documents to derive indicators of the health status of women, who are an often-overlooked population in prison structures and policies as well as in prison health surveillance.

Research to Understand Stakeholders' Prison Health Surveillance Needs and Priorities

In the context of a dearth of information in the scientific literature about stakeholders' interests for prison health surveillance, we are conducting an embedded qualitative research project using reflexive thematic analysis [17] to understand stakeholders' needs and priorities for health surveillance of people incarcerated in Canadian prisons. Through engaging with members of the Project Advisory Committee, we are including knowledge users' input in the study design and implementation, harnessing the methodological expertise of the academic partner and the lived experience and context expertise of these knowledge users [18, 19] Study participants include people with lived or current experience of incarceration, family members of people with lived experience of incarceration, people working in community organizations advocating for currently or previously incarcerated people and their health, healthcare providers and other staff from the prison authority, and academics and clinicians who work in various capacities related to prison health, with recruitment using snowball sampling starting with individuals and organizations known to members of the Project Advisory Committee. The study is ongoing, and findings will be disseminated in reports for the prison authority and publications in peer-reviewed journals.

Mapping Work to Understand Available Health-Related Data in the Prison Authority

We are exploring what health-related data are currently available in correctional and health administrative data, which could serve as health status indicators for surveillance purposes. We have reviewed the prison authority's intake assessment form to identify any variables that indicate diagnoses or other elements of health status, and reviewed the medication formulary to identify medications that are likely to indicate a specific diagnosis. We have also mapped the prison authority's EHR disease registry coding system to a standard classification system (International Classification of Disease [ICD-9]) to support comparison of health data (i.e., disorders and risk factors) for the incarcerated population with those for other Canadians.

DISCUSSION

Developing and implementing health surveillance in prisons is complex and, compared with whole population health surveillance, requires special attention and processes that are tailored to meet the particular needs and challenges of the prison context. This grant-funded project affords opportunities for dedicated, collaborative work to strengthen health surveillance in a Canadian correctional authority.

Given complex histories and at times varying interests, there is substantial value in working with diverse groups that hold a stake in prison health surveillance. In this project, we are collaborating with people with lived experience of incarceration, people involved in service delivery and advocacy for people who experience incarceration, and researchers, in addition to staff and administrators in the prison authority, and people in each of these groups are providing their perspectives regarding prison health surveillance through our research project. Recognizing the challenges involved with establishing collaborative structures and building relationships, we have found that having one partner (i.e., the academic partner in this project) facilitate the project and liaise between groups is valuable to foster ongoing collaboration.

While prison health surveillance should build on international standards for health surveillance as well as scholarly work, it should also be informed by stakeholders' needs and priorities, which may be identified through research or ongoing engagement structures. Engagement with stakeholders allows us to understand and incorporate a focus on local, regional, and national priorities that may not be represented in international standards, such as the over-incarceration of Indigenous Peoples in Canadian prisons as a consequence of colonialism and other structural factors [20], which compels a greater effort by health data collectors and analysts to take steps to counter the systemic discrimination still experienced by this historically disadvantaged population. The importance of understanding stakeholder needs notwithstanding, there are inherent challenges to data collection and reporting in the context of prisons, and prison health surveillance may be driven by what is feasible as well as acceptable from the perspectives of the prison authority and people who experience incarceration.

We recognize that the prison authority is the ultimate decision-maker regarding prison health surveillance, and that there are legal, bureaucratic, technical, and resource challenges to implementing changes to prison health surveillance systems. Project activities can contribute specific inputs to address some of these challenges and bolster ongoing work, and partnership and collaboration beyond the bounds of this project would be valuable to further strengthen prison health surveillance in this prison authority. We recommend that prison authorities and their potential partners in other jurisdictions work to develop collaborative relationships and structures to leverage expertise, identify needs and interests, and thereby enhance health surveillance, with the ultimate goal of contributing to population health.

ETHICS STATEMENT

The overall project did not require ethics review. One project component was a research project to understand stakeholders' prison health surveillance needs and priorities (described above), and we obtained institutional ethics approval for this research study (Hamilton Integrated Research Ethics Board, project #14099).

AUTHOR CONTRIBUTIONS

FK and JG developed the structures and processes described in the process model and led the activities to strengthen surveillance. NS wrote the first draft of this paper and designed the first draft of the process model figure. JL supported work through literature reviews within the various activities. IG consulted throughout work on the processes, structures, and reviews and reviewed for applicability and resonance for people with lived experience of incarceration. All authors contributed to the article and approved the submitted version.

FUNDING

The author(s) declare that financial support was received for the research, authorship, and/or publication of this article. This project is funded by the Public Health Agency of Canada's Enhanced Surveillance for Chronic Disease Program (ESCDP), arrangement #2021-HQ-000098.

CONFLICT OF INTEREST

The authors declare that they do not have any conflicts of interest.

REFERENCES

1. World Health Organization. *GHO | Health in Prisons European Database (HIPED)*. Geneva, Switzerland: World Health Organization (2019). Available from: https://apps.who.int/gho/data/node.prisons (Accessed July 12, 2022).
2. Lawrence OG, Vanchieri C, Pope A. *Ethical Considerations for Research Involving Prisoners*. Washington, DC: National Academies Press (2007). p. 1–265.
3. Binswanger IA, Maruschak LM, Mueller SR, Stern MF, Kinner SA. Principles to Guide National Data Collection on the Health of Persons in the Criminal Justice System. *Public Health Rep* (2019) 134(1_Suppl. l):34S–45S. doi:10.1177/00333549198415 93
4. Choi BCK. The Past, Present, and Future of Public Health Surveillance. *Scientifica (Cairo)* (2012) 2012:875253–26. doi:10.6064/2012/875253
5. Fazel S, Baillargeon J. The Health of Prisoners. *The Lancet* (2011) 377(9769): 956–65. doi:10.1016/S0140-6736(10)61053-7
6. Kouyoumdjian F, Schuler A, Matheson FI, Hwang SW. Health Status of Prisoners in Canada: Narrative Review. *Can Fam Physician* (2016) 62(3):215–22. Available from: https://pmc/articles/PMC4984599/ (Accessed July 11, 2022).
7. Binswanger IA, Krueger PM, Steiner JF. Prevalence of Chronic Medical Conditions Among Jail and Prison Inmates in the USA Compared With the General Population. *J Epidemiol Community Health* (2009) 63(11):912–9. doi:10.1136/jech.2009.090662
8. Verdot C, Godin-Blandeau E, Grémy I, Develay AE. Monitoring Systems and National Surveys on Prison Health in France and Abroad. *Eur J Public Health* (2015) 25(1):167–72. doi:10.1093/eurpub/cku141
9. Ahalt C, Binswanger IA, Steinman M, Tulsky J, Williams BA. Confined to Ignorance: The Absence of Prisoner Information From Nationally Representative Health Data Sets. *J Gen Intern Med* (2012) 27(2):160–6. doi:10.1007/s11606-011-1858-7
10. Statistics Canada. Surveys and Statistical Programs—National Household Survey (NHS) (2016). Available from: https://www23.statcan.gc.ca/imdb/p2SV.pl?Function=getSurvey&SDDS=5178 (Accessed January 8, 2024).
11. CDC. *Evaluating an NCD-Related Surveillance System*. Atlanta, GA: Centers for Disease Control and Prevention (2013). Available from: https://www.cdc.gov/globalhealth/healthprotection/fetp/training_modules/12/eval-surv-sys_pw_final_09262013.pdf (Accessed July 12, 2022).
12. Perrett S, Plugge E, Conaglen P, O'Moore E, Sturup-Toft S. The Five Nations Model for Prison Health Surveillance: Lessons From Practice Across the UK and Republic of Ireland. *J Public Health (Bangkok)* (2020) 42(4):e561–72. doi:10.1093/pubmed/fdz122
13. Statistics Canada. Average Counts of Offenders in Federal Programs, Canada and Regions (2023). Table 35-10-0155-01. Available from: https://www150.statcan.gc.ca/t1/tbl1/en/tv.action?pid=3510015501 (Accessed December 18, 2023).
14. Public Safety Canada. Corrections and Conditional Release Statistical Overview 2021 Annual Report (2021). Available from: https://www.publicsafety.gc.ca/cnt/rsrcs/pblctns/ccrso-2021/index-en.aspx#sec-d10 (Accessed July 16, 2024).
15. UNODC. The United Nations Standard Minimum Rules for the Treatment of Prisoners (The Nelson Mandela Rules) (2015). Available from: https://www.unodc.org/documents/justice-and-prison-reform/Nelson_Mandela_Rules-E-ebook.pdf (Accessed January 8, 2024).
16. UNODC. *The Bangkok Rules United Nations Rules for the Treatment of Women Prisoners and Non-Custodial Measures for Women Offenders With Their Commentary* (2010). Available from: https://www.unodc.org/documents/justice-and-prison-reform/Bangkok_Rules_ENG_22032015.pdf (Accessed January 8, 2024).
17. Braun V, Clarke V. *Thematic Analysis: A Practical Guide*. Thousand Oaks, CA: SAGE Publications Ltd. (2022).
18. Gagliardi AR, Berta W, Kothari A, Boyko J, Urquhart R. Integrated Knowledge Translation (IKT) in Health Care: A Scoping Review. *Implementation Sci* (2016) 11(1):38–12. doi:10.1186/s13012-016-0399-1
19. Kothari A, McCutcheon C, Graham ID. Defining Integrated Knowledge Translation and Moving Forward: A Response to Recent Commentaries. *Int J Health Pol Manag* (2017) 6:299–300. doi:10.15171/ijhpm.2017.15
20. National Inquiry Into Missing and Murdered Indigenous Women and Girls. *A Legal Analysis of Genocide Supplementary Report of the National Inquiry Into Missing and Murdered Indigenous Women and Girls* (2019). Available from: https://www.mmiwg-ffada.ca/wp-content/uploads/2019/06/Supplementary-Report_Genocide.pdf (Accessed July 16, 2024).

Copyright © 2024 Gaber, Sanya, Lawson, Grenada and Kouyoumdjian. This is an open-access article distributed under the terms of the Creative Commons Attribution License (CC BY). The use, distribution or reproduction in other forums is permitted, provided the original author(s) and the copyright owner(s) are credited and that the original publication in this journal is cited, in accordance with accepted academic practice. No use, distribution or reproduction is permitted which does not comply with these terms.
