# Supplementary material for: Healthcare considerations for aging people in prisons
Source: PLoS One. 2026 Apr 30;21(4):e0347851. doi: 10.1371/journal.pone.0347851 (PMC13132422; doi:10.1371/journal.pone.0347851)
Supplement: Attachment — Submitted filename: Manuscript Number PONE-D-25-48938R.docx [file pone.0347851.s004.docx]

**Manuscript Number PONE-D-25-48938R1**

I am content that my peer review comments from the prior manuscript have been addressed and to recommend accepting this manuscript. There is a minor issue with repetition of ‘or’ around line 105, and repetition of ‘questioning’ in the added text.
